# Bi_1−x_Eu_x_FeO_3_ Powders: Synthesis, Characterization, Magnetic and Photoluminescence Properties

**DOI:** 10.3390/nano9101465

**Published:** 2019-10-16

**Authors:** Vasile-Adrian Surdu, Roxana Doina Trușcă, Bogdan Ștefan Vasile, Ovidiu Cristian Oprea, Eugenia Tanasă, Lucian Diamandescu, Ecaterina Andronescu, Adelina Carmen Ianculescu

**Affiliations:** 1 Department of Science and Engineering of Oxide Materials and Nanomaterials, Faculty of Applied Chemistry and Materials Science, “Politehnica” University of Bucharest, Gh. Polizu Street no. 1-7, 011061 Bucharest, Romania; adrian.surdu@upb.ro (V.-A.S.); truscaroxana@yahoo.com (R.D.T.); eugenia.vasile27@gmail.com (E.T.); ecaterina.andronescu@upb.ro (E.A.); 2 Department of Inorganic Chemistry, Physical Chemistry and Electrochemistry, Faculty of Applied Chemistry and Materials Science, “Politehnica” University of Bucharest, Gh. Polizu Street no. 1-7, 011061 Bucharest, Romania; ovidiu73@yahoo.com; 3 National Institute of Materials Physics, 077125 Bucharest-Măgurele, Romania; diamand@infim.ro

**Keywords:** bismuth ferrite, sol-gel process, magnetic properties, photoluminescence properties

## Abstract

Europium substituted bismuth ferrite powders were synthesized by the sol-gel technique. The precursor xerogel was characterized by thermal analysis. Bi_1−x_Eu_x_FeO_3_ (x = 0–0.20) powders obtained after thermal treatment of the xerogel at 600 °C for 30 min were investigated by X-ray diffraction (XRD), scanning electron microscopy (FE-SEM), transmission electron microscopy (TEM), Raman spectroscopy, and Mössbauer spectroscopy. Magnetic behavior at room temperature was tested using vibrating sample magnetometry. The comparative results showed that europium has a beneficial effect on the stabilization of the perovskite structure and induced a weak ferromagnetism. The particle size decreases after the introduction of Eu^3+^ from 167 nm for x = 0 to 51 nm for x = 0.20. Photoluminescence spectroscopy showed the enhancement of the characteristic emission peaks intensity with the increase of Eu^3+^ concentration.

## 1. Introduction

Among various multiferroic compounds, bismuth ferrite (BiFeO_3_) stands out because it is one of the few magnetic ferroelectrics at room temperature. Therefore, there has been intensive research in the past decades to make it useful in practical applications. There are certain issues that are still open in what concerns voltage-induced changes, the possibility of reading magnetic data or the mechanism of magnetoelectric coupling, and whether it may be controlled [1]. Besides, extensive studies search for the possibility of using BiFeO_3_ based materials for applications such as actuators, transducers, magnetic field sensors, information storage devices, optical imaging, photocatalysis, or gas sensors [2,3,4,5,6]. Recently, BiFeO_3_ nanopowders were found to exhibit catalytic activity for doxorubicine degradation [7].

The antiferromagnetic structure in BiFeO_3_ is quite complex, usually being considered as a G-type with a spiral spin arrangement (about 62 nm wavelength), due to the interplay between exchange and spin-orbit coupling interactions involving Fe ions. There are several strategies to enhance its magnetic properties, including chemical modifications, or control of morphology and structure. In terms of morphologies, BiFeO_3_-based nanostructures exhibit increased magnetization than the corresponding bulks, due to the perturbation of the helimagnetic order by structural peculiarities (e.g., local defects) or the specific size of nanoparticles [8,9,10,11,12].

Another way to modify the magnetic structure consists in replacing Bi by rare earth ions, based on the fact that in the perovskite-like structure, the superexchange interaction between the localized RE*4f* and Fe*3d* electrons may play an important role. The effect of several rare-earth dopants/solutes, as Ho [13,14], Sm [15,16], La [17,18], Dy [19], Gd [20], or Nd [21] on the properties of bismuth ferrite have been investigated. There are some works which described the effect of Eu^3+^ used as *A* site solute on the characteristics of bismuth ferrite powders prepared by various non-conventional techniques, such as hydrothermal process [22], ball milling [23], or different variants of the sol-gel methods [24,25,26], etc. Even if the magnetic behavior of these powders was extensively analyzed, however no data regarding other properties as photoluminescence were reported.

The aim of this work is to study the influence of europium addition on the phase purity, crystal structure, morphology, magnetic behavior, and optical properties of Bi_1−x_Eu_x_FeO_3_ powders (x = 0; 0.05; 0.10; 0.15; 0.20) prepared by the sol-gel route. In order to be able to assess only the contribution of Eu substitution on the *A*-site of the perovskite structure, all the processing parameters were constantly maintained. 

## 2. Materials and Methods

Synthesis of Bi_1−x_Eu_x_FeO_3_ powders (x = 0; 0.05; 0.10; 0.15; 0.20) was carried out through sol-gel route. All the solvents and chemicals were of analytical grade and used without further purification. The precursor solution was prepared by dissolution of Bi(NO_3_)_3_·5H_2_O (Sigma Aldrich, St. Louis, MO, USA ≥98%), Eu(NO_3_)_3_·5H_2_O (Sigma Aldrich, 99.9%) and Fe(NO_3_)_3_·9H_2_O (Aldrich, 99.99%) in stoichiometric ratios in acetic acid solution (Honeywell Fluka, Wabash, IN, USA ACS Reagent, ≥99.7%). A transparent brownish red sol resulted after the complete dissolution (≈ 1 h) of the nitrates. The sol was stabilized with 2-methoxyetanol which was added in a 1:1 volume ratio with respect to acetic acid. The amounts of precursors are summarized in Table 1. After 1 h mixing at 400 rpm, the temperature was set to 80 °C and the sol was kept under magnetic stirring at this temperature for 12 h until a gel was obtained. Gel drying was carried out in a forced convection oven (Memmert Universal Oven U, Schwabach, Germany) in air at 120 °C for 12 h to obtain the xerogels. The precursor powders were heat treated in air at 600 °C with a soaking time of 30 min, a heating rate of 5 °C/min and then were slowly cooled at the normal rate of the oven (CWF 1200, Carbolite Gero, Hope Valley, England).

Thermal behavior of the precursor powders was investigated by differential scanning calorimetry–thermogravimetry (DSC-TG) analyses carried out with a TG 449C STA Jupiter (Netzsch, Selb, Germany) thermal analyzer. Samples were placed in alumina crucible and heated with 10 °C/min from room temperature to 900°, under dried air flow of 20 mL/min.

Room temperature X-ray diffraction (XRD) measurements were performed to investigate the phase purity and structure of the (Bi,Eu)FeO_3_ powders. For this purpose, an Empyrean diffractometer (PANalytical, Almelo, The Netherlands), using Ni-filtered Cu-Kα radiation (λ = 1.5418 Å) with a scan step increment of 0.02° and a counting time of 255 s/step, for 2θ ranged between 20–80°was used. Lattice parameters were refined by the Rietveld method [27], using the HighScore Plus 3.0e software (PANalytical, Almelo, The Netherlands). After removing the instrumental contribution, the full-width at half-maximum (FWHM) of the diffraction peaks can be interpreted in terms of crystallite size and lattice strain. A pseudo-Voigt function was used to refine the shapes of the BiFeO_3_ peaks.

The local order and the cation coordination in the calcined powders were studied by Raman spectroscopy carried out at room temperature, using a LabRAm HR Evolution spectrometer (Horiba, Kyoto, Japan). Raman spectra were recorded using the 514 nm line of an argon ion laser, by focusing a 125 mW beam of a few micrometer sized spots on the samples under investigation.

Mössbauer spectroscopy ICE Oxford Mössbauer cryomagnetic system (WissEL, Mömbris, Germany) was used to analyze the state of iron ions in the perovskite lattice. The system was equipped with a 10 mCi ^57^Co(Rh) source and the velocity was calibrated using a α-Fe standard foil.

Morphology and crystallinity degree of the (Bi,Eu)FeO_3_ particles were investigated by scanning electron microscopy operated at 30 kV (Inspect F50, FEI, Hillsboro, OR, USA) and transmission electron microscopy operated at 300 kV (Tecnai^TM^ G2 F30 S-TWIN, FEI, Hillsboro, OR, USA). The average particle size of the (Bi,Eu)FeO_3_ powders was estimated from the particle size distributions, which were determined using the OriginPro 9.0 software (OriginLab, Northampton, MA, USA) by taking into account size measurements on ~100 particles performed by means of the software of the electron microscopes (ImageJ 1.50b, National Institutes of Health and the Laboratory for Optical and Computational Instrumentation, Madison, WI, USA) in the case of SEM, and Digital Micrograph 1.8.0 (Gatan, Sarasota, FL, USA) in the case of transmission electron microscopy (TEM).

Vibrating sample magnetometry (7404-s VSM, LakeShore, Westerville, OH, USA) was used in order to investigate the magnetic behavior of the processed powders. Hysteresis loops were recorded at room temperature with an applied field up to 15 kOe, with increments of 200 Oe and a ramp rate of 20 Oe/s.

The fluorescence spectra were recorded with a LS 55 spectrometer (Perkin Elmer, Waltham, MA, USA) using an Xe lamp as a UV light source, at ambient temperature, in the range 350–650 nm, with all the samples in solid state. The measurements were made with a scan speed of 200 nm/min, excitation and emission slits of 10 nm, and a cut-off filter of 350 nm. An excitation wavelength of 320 nm was used.

## 3. Results

### 3.1. Thermal Behavior of the Precursor Powders

**Figure 1:**
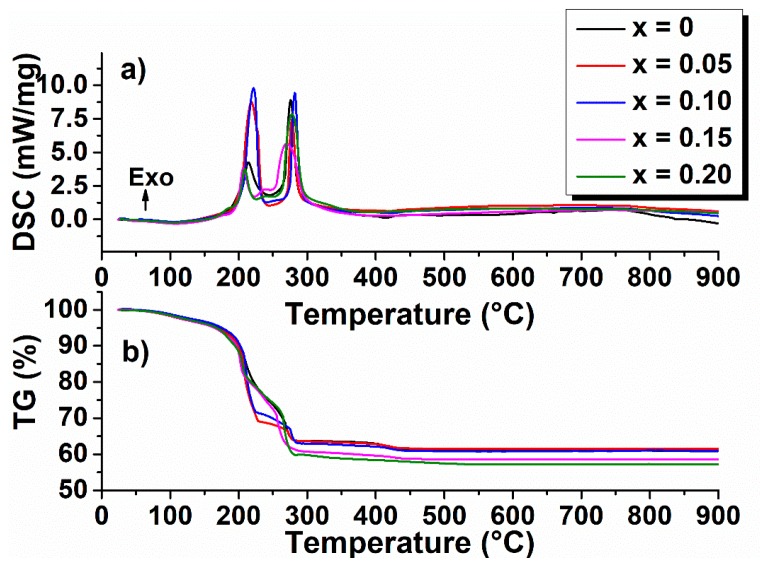
(**a**) Differential scanning calorimetry (DSC) and (**b**) thermogravimetry (TG) curves of Bi_1−x_Eu_x_FeO_3_ xerogels.

The TG-DSC curves of the Bi_1−x_Eu_x_FeO_3_ xerogels are shown in Figure 1. The peaks corresponding to the exothermic effects and associated mass loss are illustrated in Table 2.

Thermal analysis reveals four step decomposition in the case of (Bi,Eu)FeO_3_ powders with x = 0, x = 0.05, x = 0.10, and five step decomposition for the samples with x = 0.15 and x = 0.20. The first step decomposition at 103–108° was attributed to dehydration of the xerogels. 

The second decomposition step (110–230 °C) associated with exothermic reactions with the highest mass loss, between 18.2% and 26.5% for the selected compositions, correspond to decarboxylation of acetic acid and decomposition of small groups such as NO_3_^−^. For the powders with x = 0.15 and x = 0.20, this reaction takes place in two steps, one at 206.7 °C and 208.7 °C, respectively, and the other at 239.2 °C and 240.3 °C, respectively [28].

The exothermic effect at 270–282 °C could be assigned to the collapse of the gel network and combustion of most organic materials. A small weight loss ≈2.5% occurring up to 430 °C corresponds to the end of CO_2_ release [28].

### 3.2. Phase Composition and Structure of the (Bi,Eu)FeO_3_ Powders

**Figure 2:**
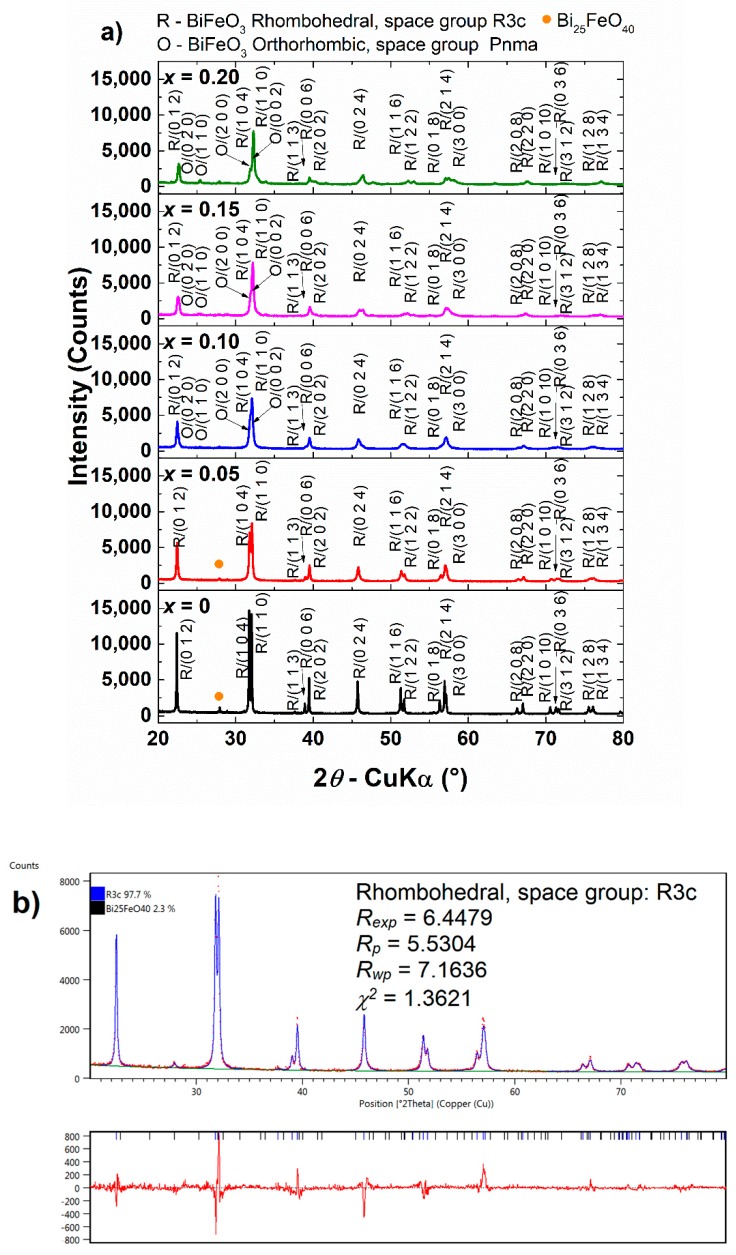

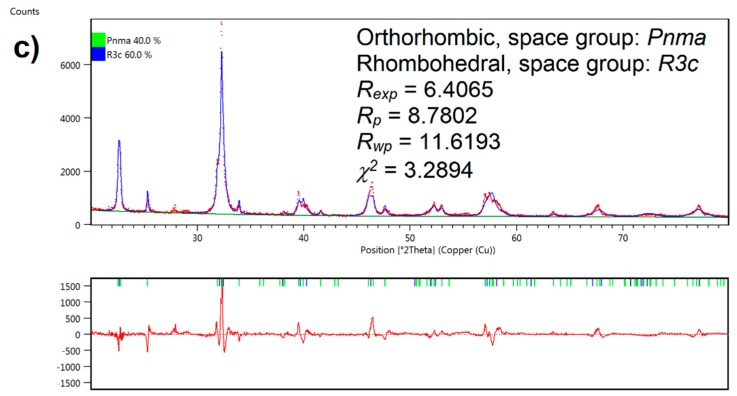
(**a**) X-ray diffraction (XRD) patterns of Bi_1−x_Eu_x_FeO_3_ calcined powders, (**b**,**c**) Rietveld refined patterns for x = 0.05 and x = 0.20.

The room-temperature XRD patterns of Bi_1−x_Eu_x_FeO_3_ powders are illustrated in Figure 2. The profiles of the peaks indicate a high crystallinity. A rhombohedral perovskite structure with space group R3c was indexed for the powders with x ≤ 0.10 [23]. A small amount of Bi_25_FeO_40_ sillenite phase is also detected for these compositions. Upon increasing the substitution ratio, the secondary phase diminishes until vanishing, which proves the beneficial effect of Eu^3+^ in what concerns the stabilization of the perovskite phase. All the reflections corresponding to the major perovskite phase are shifted to higher values of the diffraction angle when x is increased. Besides, in the case of the compositions with x ≥ 0.15 it may be observed that the (012) peak is split and a supplementary interference occurs at 2θ ≈ 34°. These are arguments that suggest Eu^3+^ ions have substituted Bi^3+^ in the BiFeO_3_ lattice and that a phase transition from rhombohedral R3c (α phase) to orthorhombic Pnma (β phase) crystal symmetry has occurred [29,30]. 

Rietveld refinement was performed in order to accurately determine the phase composition and structure of the powders. For the specimens with x ≥ 0.15, the best fit to data was obtained when using a mixture of rhombohedral R3c and orthorhombic Pnma polymorphs. The quality of the fits is indicated by the agreement indices obtained from Rietveld refinement (Table 3).

**Figure 3:**
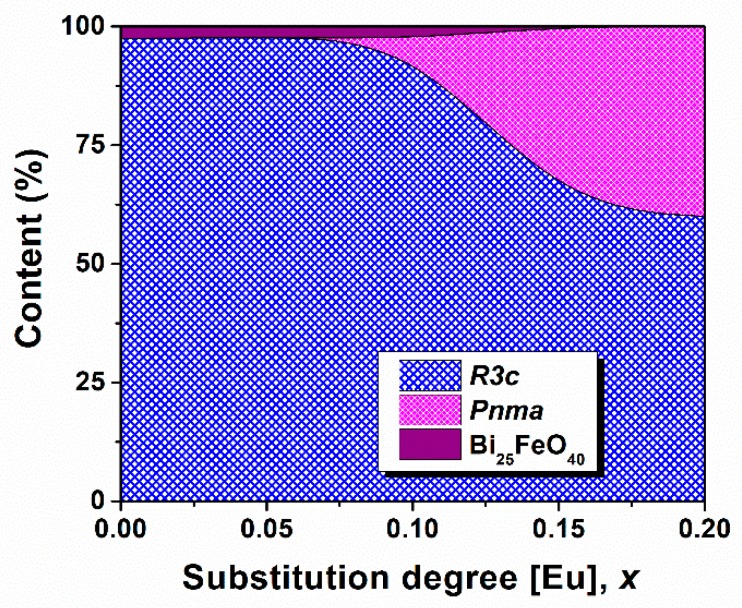
Phase composition evolution in Bi_1−x_Eu_x_FeO_3_ calcined powders.

The phase composition evolution versus Eu^3+^ substitution degree is shown in Figure 3. For x ≥ 0.15, the Bi_25_FeO_40_ secondary phase vanishes in the limit of detection of X-ray diffraction. Stabilization of the perovskite phase is also accompanied by rapid polymorph transition. When increasing x from 0.10 to 0.15, phase composition changes from 97.4% R3c bismuth ferrite and 2.6% sillenite in the secondary phase to 62% R3c bismuth ferrite polymorph and 40% Pnma bismuth ferrite polymorph, respectively. These results are in good agreement with those reported by Iorgu et al. [31] and Khomchenko et al. [32] who also found a second orthorhombic polymorph in their Eu-substituted bismuth ferrite, with x ≥ 0.10 obtained by combustion method and solid state reaction, respectively.

**Figure 4:**
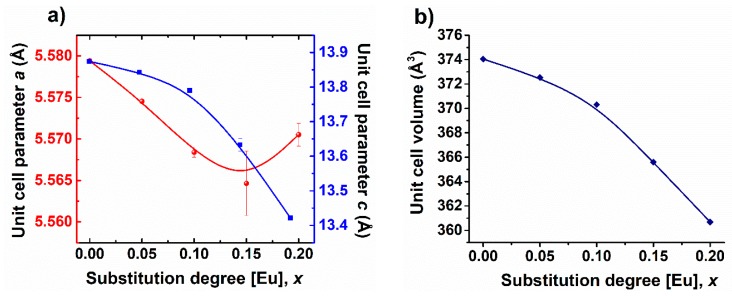
(**a**) Unit cell parameters corresponding to R3c polymorph, and (**b**) unit cell volume for Bi_1−x_Eu_x_FeO_3_ calcined powders.

Unit cell parameters and cell volume (Figure 4) decrease with the increasing amount of Eu solute. This, together with the phase transition, is supported most likely by the smaller ionic radius of Eu^3+^ (1.07 Å) than that of Bi^3+^ (1.17 Å) [33]. 

**Figure 5:**
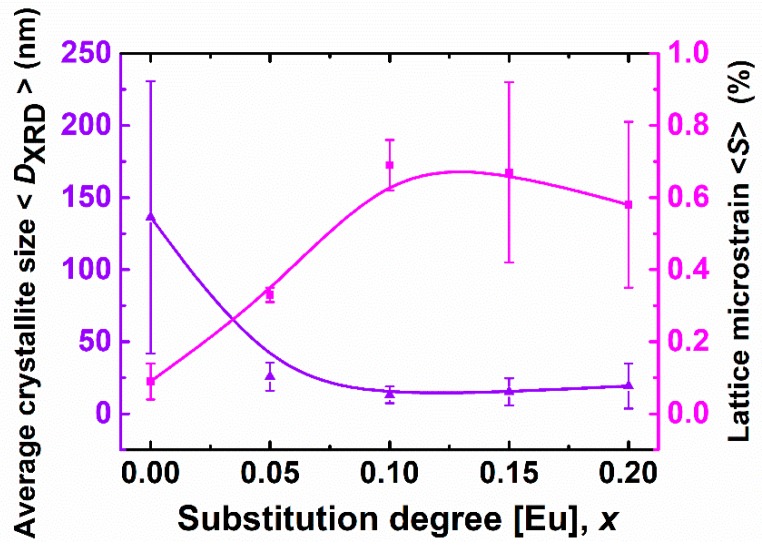
Average crystallite size and lattice microstrain for Bi_1−x_Eu_x_FeO_3_ calcined powders.

As expected, the formation of (Bi,Eu)FeO_3_ solid solutions drives to the decrease of the crystallite size and the concurrent increase of the internal microstrains (Figure 5).

Raman spectroscopy is a powerful technique, which is sensitive to structural phase transitions and it has been carried out to further support the Rietveld analysis of the XRD patterns. The active Raman modes of the BiFeO_3_ solid solutions with rhombohedral R3c structure may be summarized using the irreducible representation of Γ_Raman, R3c_ = 4A_1_ + 9E [34,35,36]. 

**Figure 6:**
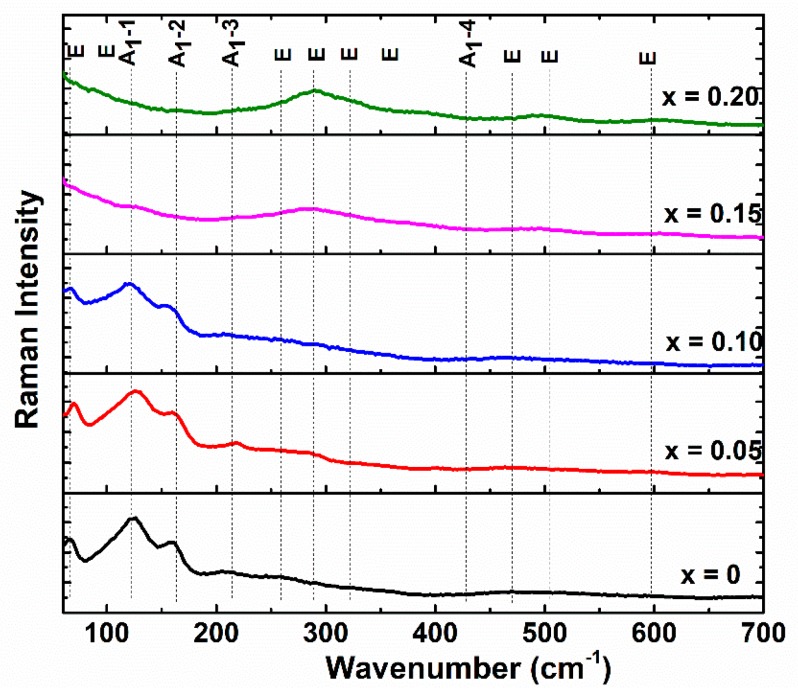
Typical Raman scattering spectra of Bi_1−x_Eu_x_FeO_3_ calcined powders.

In the present study, for the powders with lower Eu content (x ≤ 0.10), the modes A_1_-1 and A_1_-2, attributed to Bi-O bonds shift to higher-frequency region. This may be explained by the partial substitution of Bi^3+^ with Eu^3+^ because the frequency of the mode is inversely proportional to the mass, M, at *A*-site. Since the mass of Eu is about 27% lower than the mass of Bi, substitution will induce the shift in the frequency of vibration of the modes, which is consistent to the data presented in Figure 6. When x increases from 0.10 to 0.15, the most significant feature in the Raman spectra is that A_1_-1 and A_1_-2 modes almost vanish and severely broaden, while the E mode at ≈290 cm^−1^ shifts to a higher frequency and increases in intensity. Such peak has been reported for orthorhombic rare earth ferrites and can be assigned to A_g_ mode [37]. The further increase of x from 0.15 to 0.20 indicate a visible distortion of FeO_6_ octahedra, which is evidenced by the increase of intensity of the 500 and 600 cm^−1^ modes [38]. All the discussed features are arguments that Eu^3+^ is incorporated on the Bi site of the perovskite lattice of BiFeO_3_ forming solid solutions, and that when the substitution degree exceeds the value of 0.15, using the processing parameters in the present work, it induces a structural phase transition from rhombohedral to orthorhombic symmetry.

**Figure 7:**
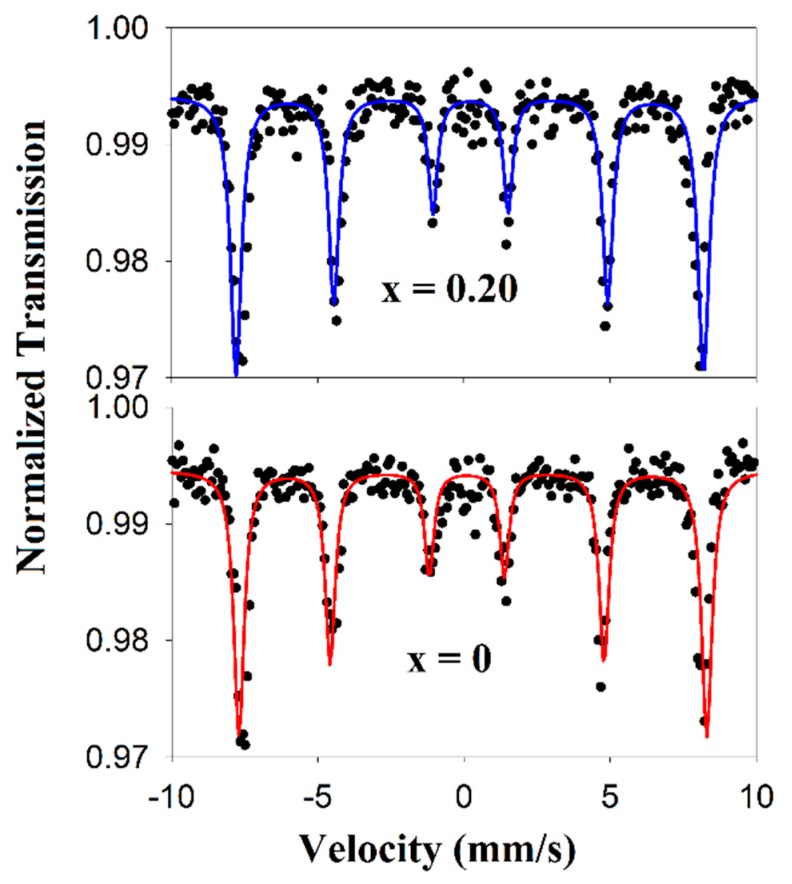
Room temperature ^57^Fe Mössbauer spectra of powders with x = 0 and x = 0.20.

The ^57^Fe Mössbauer spectra for the selected compositions with x = 0 and x = 0.20 were recorded at room temperature. Results show that the spectra corresponding to the investigated samples present hyperfine magnetic sextet (Figure 7). 

The refining of the spectra under the assumption of the Lorentzian shape of the Mössbauer line allowed obtaining of the characteristic parameters: isomeric shift (IS), quadrupole splitting (ΔEq) and hyperfine field (H_hf_), which are presented in Table 4.

The values of IS and ΔEq prove that Fe occupies only the B-site in the perovskite structure and correspond to high-spin Fe^3+^ ions.

Upon introduction of Eu^3+^ in the lattice, ΔEq switches from positive values (0.179 mm/s) in the case of x = 0 to negative values (−0.054 mm/s) in the case of x = 0.20. This means that the electric field gradient is drastically changed by the substitution and may be assigned to structural phase transition from rhombohedral to orthorhombic symmetry, as seen for Bi_1−x_Dy_x_FeO_3_ nanoparticles obtained by Qian et al. [39] 

In what concerns the H_hf_, the substitution of Bi^3+^ with Eu^3+^ does not affect the obtained values nor the charge density reflected in the IS parameter which remains constant. All Mössbauer parameters are in good agreement with those obtained by Prado-Gonjal et al. for microwave-assisted hydrothermal processed BiFeO_3_ powders [40].

### 3.3. Morphology

**Figure 8:**
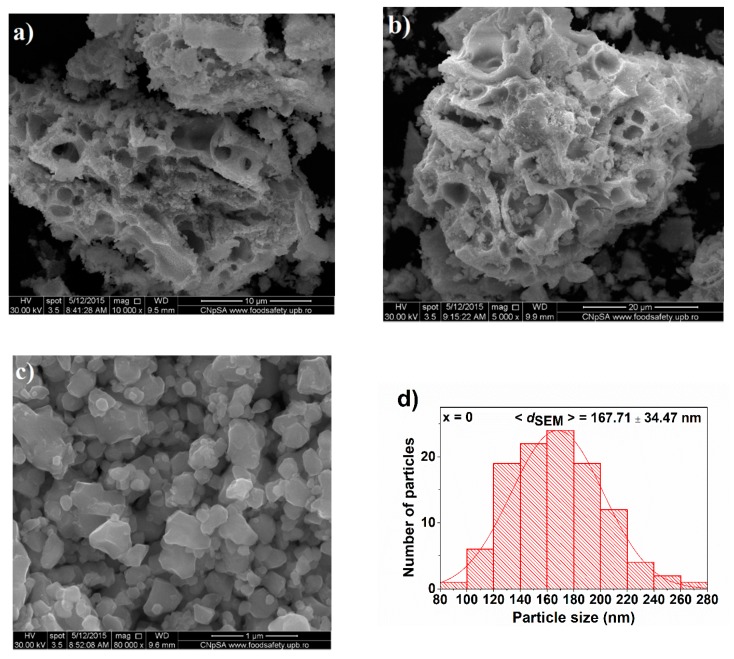

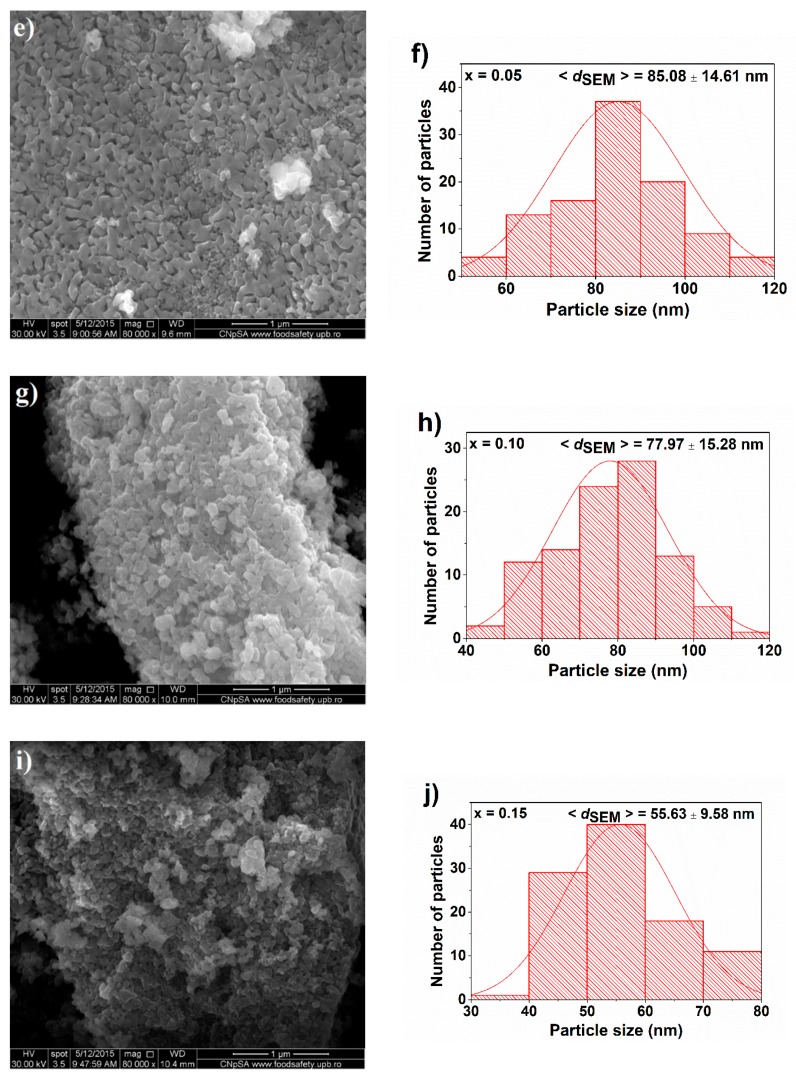

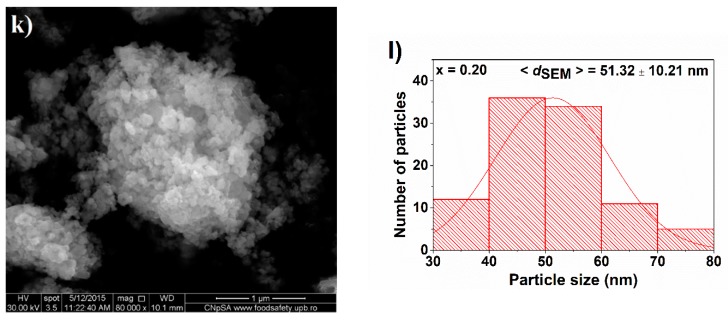
FE-SEM images showing the morphology and corresponding histograms for particle size distribution of Bi_1−x_Eu_x_FeO_3_ powders: (**a**,**b**) General view for x = 0 and x = 0.10, (**c**,**d**) x = 0: (**c**) detail, (**d**) particle size distribution, (**e**,**f**) x = 0.05: (**e**) detail, (**f**) particle size distribution, (**g**,**h**) x = 0.10: (**g**) detail, (**h**) particle size distribution, (**i**,**j**) x = 0.15: (**i**) detail, (**j**) particle size distribution, (**k**,**l**) x = 0.20: (**k**) detail, (**l**) particle size distribution,.

Scanning electron microscopy (FE-SEM) images depicting the morphology and the particle size distribution of the calcined (Bi,Eu)FeO_3_ powders are shown in Figure 8. A general view of two selected compositions, x = 0 (Figure 8a) and x = 0.10 (Figure 8b), illustrate porous networks with pores in the micrometer and submicrometer range, which were formed after heat treatment of the precursor gels. The walls of the pores are dense and consist of agglomerated particles as it may be seen in the detail in the images of Figure 8c,e,g,i,k. In each case, the particles exhibit polyhedral shapes and as x increases the particles tend to have a more rounded aspect. Moreover, for ternary compositions, a tendency toward coarsening was observed. In what concerns the particle size, one can see a decrease after the introduction of Eu^3+^ as a substituent in the perovskite lattice from 167 nm for x = 0 to 85 nm for x = 0.05, which becomes even more evident for the compositions where the polymorphic transformation occurs (x = 0.15 and x = 0.20). In the latter case, the particle size decreases from 78 nm for x = 0.10 to 56 nm for x = 0.15. This kind of effect is consistent with other studies regarding substituted BiFeO_3_ particles prepared by various techniques [24,26,31,41]. Moreover, Dai et al. explained this in the case of (Eu, Ti) co-substituted ceramics as a result of suppression of oxygen vacancies by the solutes, which slows oxygen ion motion and, consequently, grain growth rate [42]. The particle size distribution is unimodal (Figure 8d,f,h,j,l) and becomes narrower as the solute concentration increases. Thus, in the BiFeO_3_ sample, the unimodal distribution show 20%–25% of nanoparticles in the size range of 140–180 nm. Besides, the influence of the addition of Eu^3+^ on the size and particle size distribution should be noted. The introduction of 5% Eu^3+^ results in the particle size distribution shown in Figure 8f. The entire particle size distribution is between 50 and 120 nm, with a maxima at 80–90 nm, which represents a proportion of 35%. Actually, all the nanoparticles present sizes below those characteristic to BiFeO_3_ (80–280 nm). The slowing particle growth effect of europium is better observed when its concentration in the perovskite solid solution increases. Thus, for x = 0.10, even if 30% of the nanoparticles correspond to the size range of 80–90 nm, the unimodal distribution is asymmetric due to the increase of the ratio of nanoparticles in the range size of 50–80 nm. More obvious contribution of the solute is shown in the case of x = 0.15 and x = 0.20, where one can observe that 35%–40% of the nanoparticles are in the size range of 50–60 nm and, respectively, 40–60 nm. The measurements of the sizes and the corresponding distributions from FE-SEM data illustrate a clear influence of the Eu^3+^ solute on the reduction of the particles size, as well as on the narrowing of the particle size distribution with the increase of the substitution rate.

**Figure 9:**
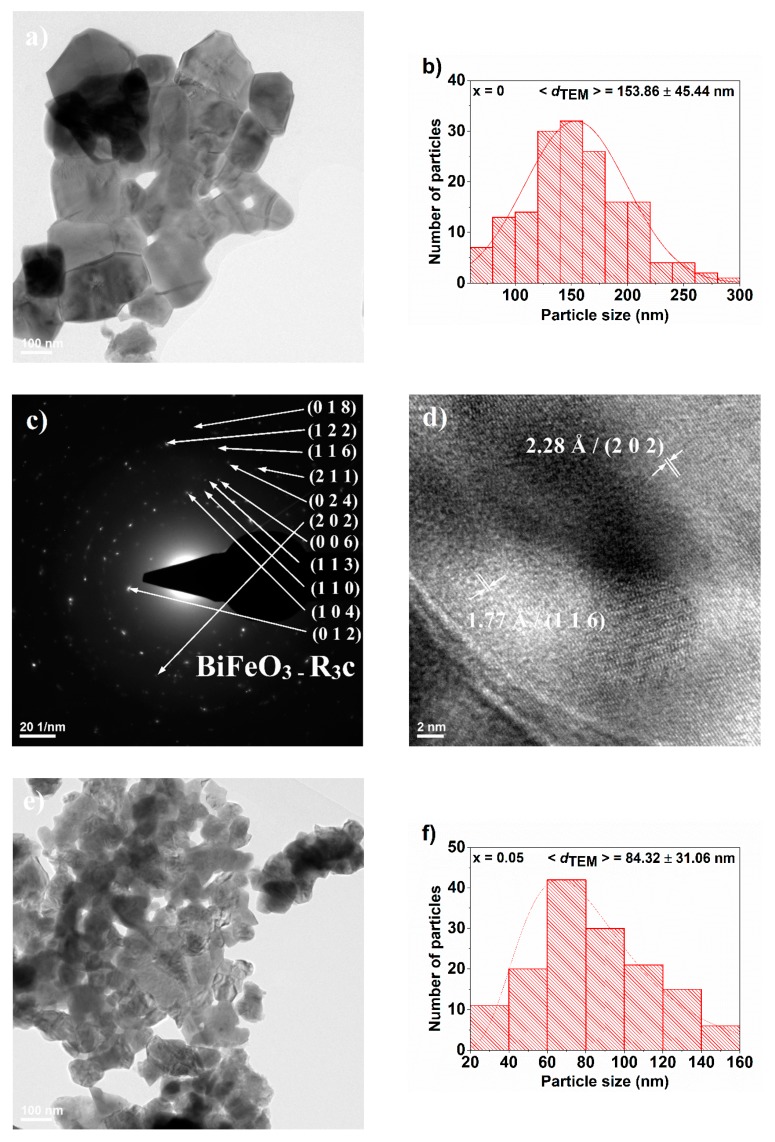

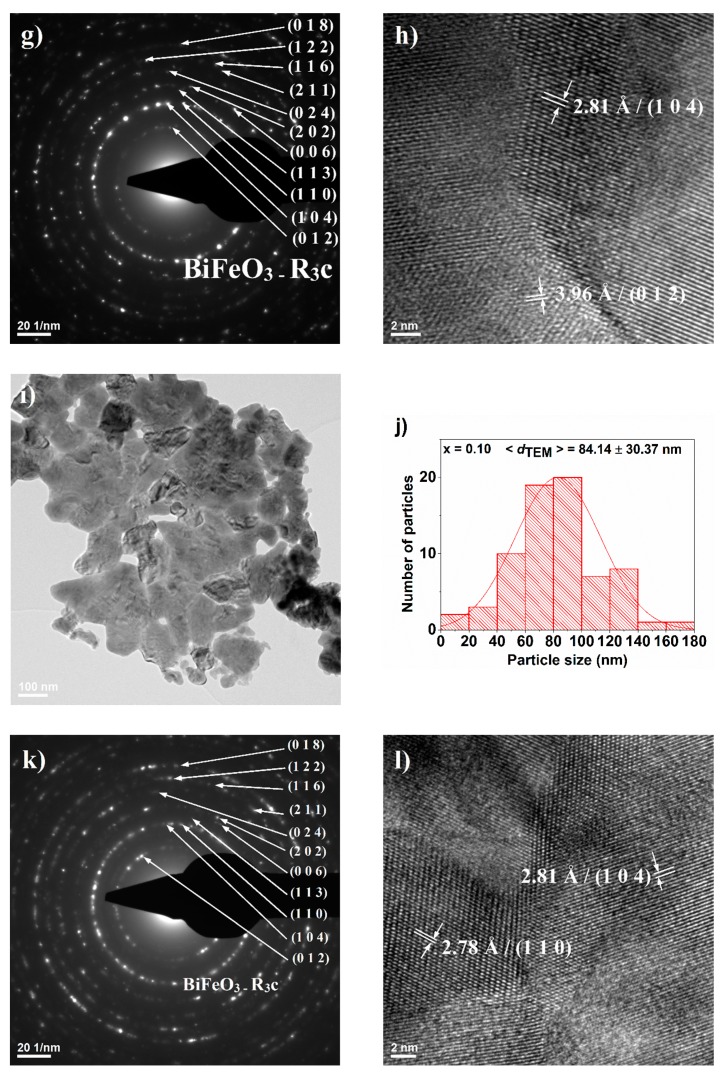

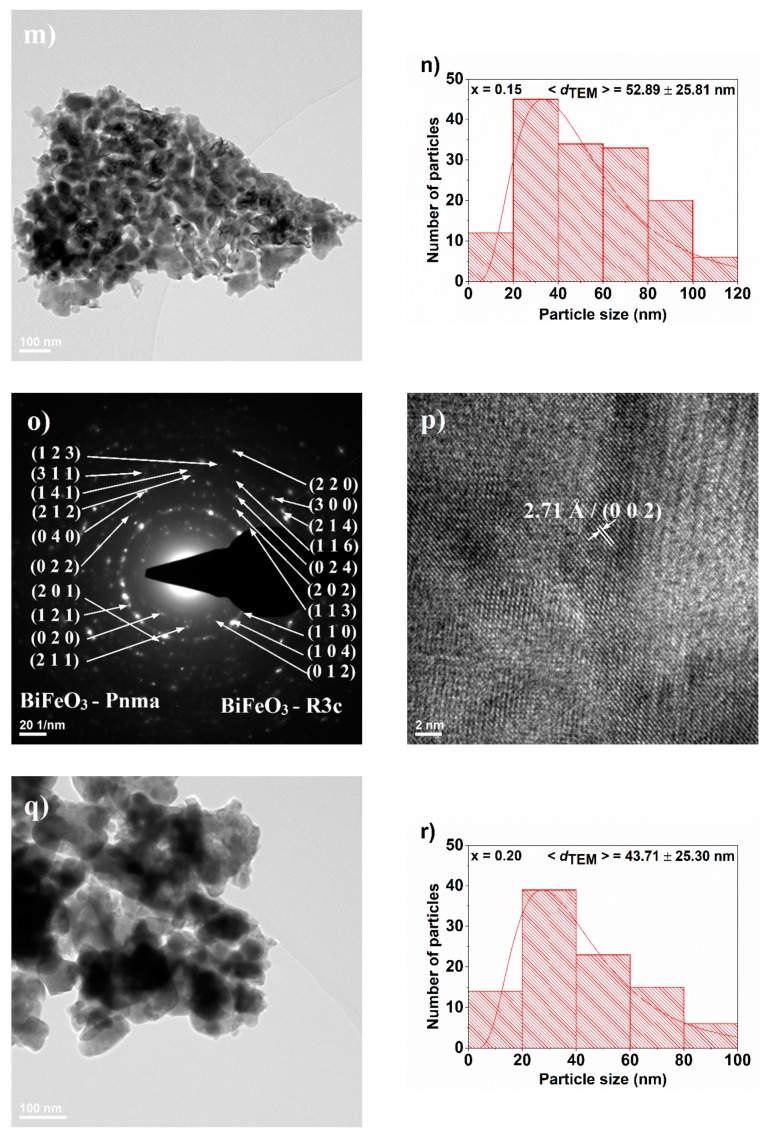

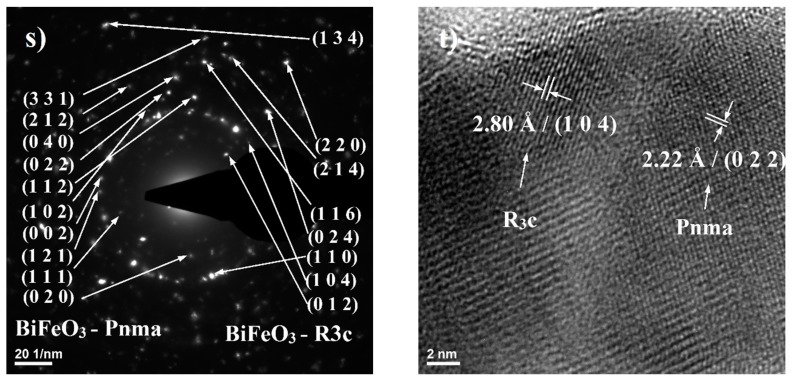
(**a**,**e**,**i**,**m**,**q**) Bright field TEM images, (**b**,**f**,**j**,**n**,**r**) particle size distributions, (**c**,**g**,**k**,**o**,**s**) Selected area electron diffraction patterns, and (**d**,**h**,**l**,**p**,**t**) High resolution TEM images corresponding to Bi_1−x_Eu_x_FeO_3_ powders for: x = 0 (**a**–**d**), 0.05 (**e**–**h**), 0.10 (**i**–**l**), 0.15 (**m**–**p**) and 0.20 (**q**–**t**), respectively.

TEM investigations sustain FE-SEM observations. The coarsening of the particles is observed better in Bright-field TEM images (Figure 9a,e,i,m,q) by means of necks at the particles limits. Particle size distributions (Figure 9b,f,j,n,r) are similar to those measured from FE-SEM images, as the small differences are in the limits of the standard deviation. Morphology evolution with increasing Eu^3+^ solute degree is similar to that reported by Bahraoui et al. who synthetized Bi_1−x_Eu_x_FeO_3_ powders by the sol-gel method with calcination treatment at 500 °C for 24 h, but the average particle size is almost four times higher [26]. This shows that although the time of heat treatment at 600 °C was relatively short (30 min), the temperature has a stronger influence on the particle size growth.

The powders show a high crystallinity degree as assessed from the selected area electron diffraction (SAED) patterns (Figure 9c,g,k,o,s), which consist of well-defined diffraction spots arranged in concentric diffraction rings. For the pure BiFeO_3_ powder (x = 0), the diffraction rings are less visible due to the fact that both crystallite size and particle size are situated in the submicrometer scale and because the coarsening process may induce some preferential orientations of the aggregated particles. In the case of the samples with higher Eu^3+^ content (x = 0.15 and x = 0.20), the patterns are more complicated because of the coexistence of rhombohedral and orthorhombic polymorphs which are homogeneously distributed. 

High resolution transmission electron microscopy (HR-TEM) investigations reveal long-range highly ordered fringes with spacing at 2.28 Å and 1.77 Å corresponding to the (2 0 2) and (1 1 6) crystalline planes of the rhombohedrally-distorted perovskite structure in the case of x = 0. For x = 0.05 and x = 0.10, there were also identified the crystallographic planes specific to rhombohedral polymorphs (Figure 9f,h). In the case of x = 0.20, both polymorphs were identified in the same particles consisting of multiple crystallites. It is worth mentioning that the substitution also induces the forming of polycrystalline particles, which is also evidenced in the HR-TEM images. 

**Figure 10:**
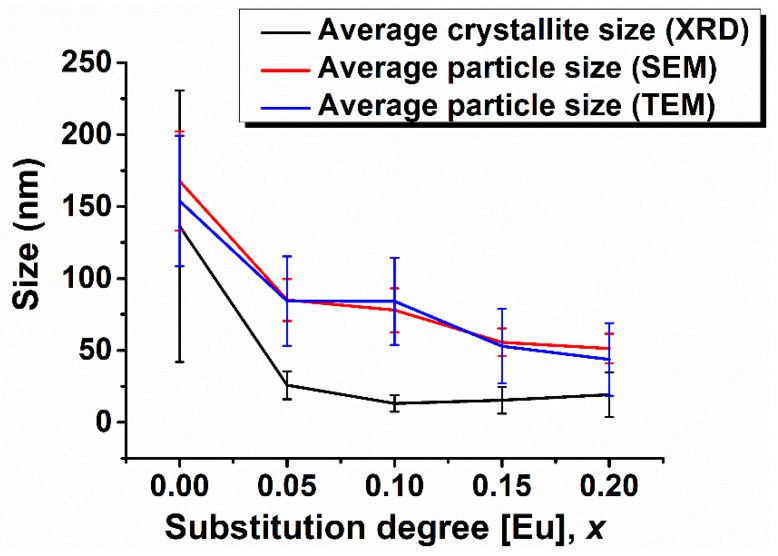
Comparison between average crystallite size determined from XRD data and average particle size measured from SEM and TEM images.

In order to have a better understanding of the nature of the particles, in Figure 10 a comparison between average crystallite size determined from XRD data and average particle size measured on SEM and TEM images was depicted. In the case of unsubstituted BiFeO_3_ particles, the three values are almost equal. Slightly differences that occur are in the range of standard deviation. This means that in this case, the particles are single crystals. Interestingly, when comparing the values obtained for Eu-substituted BiFeO_3_ compositions, one can observe that the values for the average nanoparticle size determined from SEM and TEM investigations are very close, whereas the average crystallite size presents at most a half value of the average particle size, proving that for x ≥ 0.05, the particles are polycrystalline and consist of two or more crystallites, which sustains the HR-TEM observations.

### 3.4. Magnetic Behavior

**Figure 11:**
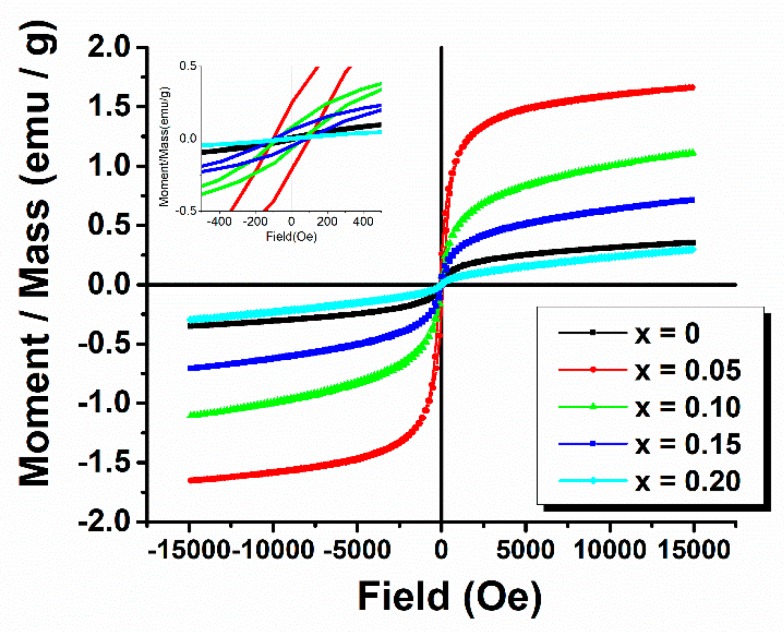
M-H hysteresis loops for Bi_1−x_Eu_x_FeO_3_ powders: Inset showing the low-field M = f(H) dependence.

Figure 11 and Table 5 show the room-temperature M = f(H) hysteresis loops up to 15,000 Oe, and M_s_, M_r_, and H_c_ parameters of the Bi_1−x_Eu_x_FeO_3_ powders. 

It can be observed that the sample with x = 0 shows a continuous linear increase of magnetization versus magnetic field which suggests the presence of the antiferromagnetic phase, involving relative low exchange integrals in order to progressively reorient the spins along the field direction. 

However, at very low fields, there is a much faster variation of saturation magnetization of 0.3529 emu/g and a coercive field of 51.290 Oe. Unlike this sample, in the case of the samples with 0.05 ≤ x ≤ 0.15, a weak ferromagnetic behavior, with a saturation magnetization of 1.6570 emu/g for x = 0.05, 1.1089 emu/g for x = 0.10 and 0.7113 emu/g for x = 0.15 is present. A possible conclusion of these aspects is that Eu content influences the spin spiral structure, most likely by a perturbation of the superexchange interactions between localized Eu*4f* and Fe*3d* electrons. 

At the maximum substitution degree studied in this work, the magnetic behavior shows a decrease in M_s_, M_r,_ and H_c_ compared to pristine BiFeO_3_ particles, suggesting that increasing Eu content above x = 0.15 does not improve the magnetic behavior of the particles. This suggests that the presence of the orthorhombic Pnma polymorph affects the magnetic order.

### 3.5. Photoluminescence Properties

BiFeO_3_ is an interesting optical material, which shows promising applications in photocatalysts and photoconductive devices. Thus, photoluminescence spectroscopy was used to study the optical property of Bi_1−x_Eu_x_FeO_3_ nanoparticles.

**Figure 12:**
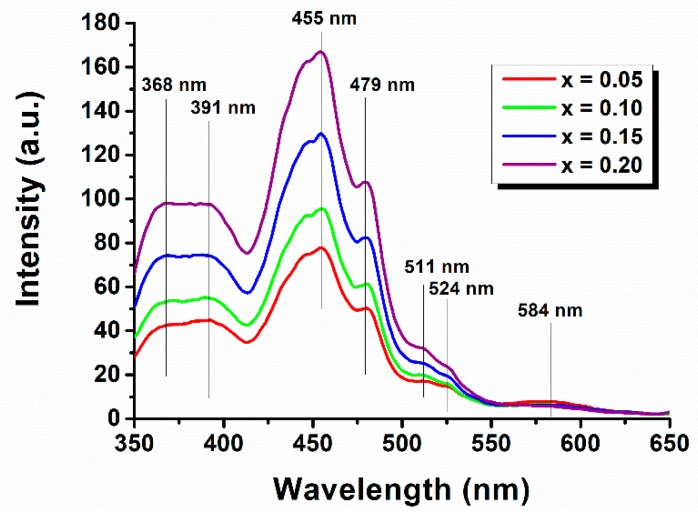
Fluorescence spectra for Bi_1−x_Eu_x_FeO_3_ powders.

During synthesis there are generated several deep and shallow oxygen vacancies and surface defects that introduce localized electronic levels in-band [43]. Therefore, the PL spectra (Figure 12) are complex and present more than a single peak from band-to-band transition. 

The most intense blue emission peak at the wavelength of 455 nm (2.72 eV) originates from self-activated centers in the synthesized nanoparticles [44,45]. The emission peak is broad and asymmetric, with a clear overlap with the peak from 479 nm (2.58 eV). This indicate the existence of another transition below the conduction band, due to the presence of defects in grain boundaries or oxygen vacancies, usually referred as near-band edge (NBE) transition [45,46,47].

In the blue-green region there are further shoulders at the 511–524 nm range which can be attributed to oxygen vacancies and a small, but broad peak in the range of 569–584 nm which has an unknown origin [48]. These peaks are usually referred as defect-level emissions (DLE).

The intensity of emission peaks increases with the increase of the solute content from the Eu^3+^-doped BiFeO_3_ with x = 0.05 to x = 0.20. This behavior cannot be explained in terms of the difference in nanoparticles dimensions, taking into account that the 5% and 10%-doped samples, as well as the 15% and 20%-doped powders exhibit roughly similar sizes.

In the first instance, for 5% Eu^3+^-doped sample, the photo-generated electron-hole pairs present a lower recombination rate, which leads to lower intensity of emission peaks. For the next three samples the increase of luminescent emission with the europium amount could be related to a higher concentration of surface defects as new crystalline phase is formed. These defects can contribute to the capture of photo-generated electrons, to produce excitons, which will enhance the emission intensity. A similar behavior was reported for Sn^4+^/Gd^3+^ or Mn^2+^-doped BiFeO_3_ samples [49,50].

In the 550–650 nm range there are no peaks that can be assigned to Eu^3+^ ions emission spectrum, indicating either a masking effect from BiFeO_3_ luminescence or, simply, a quenching of europium fluorescence. This effect was also observed for other rare earth ions used as soluted for bismuth ferrite [49,51,52].

## 4. Conclusions

Bi_1−x_Eu_x_FeO_3_ powders were prepared by the sol-gel method. XRD and Raman spectroscopy investigations indicated phase-pure particles and a structural phase transition for x ≥ 0.15 when using the processing parameters presented in the present work. Mössbauer spectroscopy showed only the presence of Fe^3+^ and a hyperfine magnetic sextet. FE-SEM and TEM analysis evidenced obtaining submicron-sized single-crystal particles for pure BiFeO_3_ composition, and polycrystalline nanoparticles in the case of Eu^3+^-substituted powders. The most pronounced ferromagnetic behavior was observed for Bi_0.95_Eu_0.05_FeO_3_ composition, which exhibited a saturation magnetization of 1.65 emu/g and a coercitive field of 100 Oe, which occurs, most likely, by a perturbation of the superexchange interactions between localized Eu*4f* and Fe*3d* electrons. This work shows a possibility to tailor magnetic behavior of bismuth ferrite using rare earth metal solute on the *A*-site of the perovskite structure. The luminescence emission increases with the increase of the Eu^3+^ content, but the quenching of the fluorescence specific to europium ions seems to be induced by a masking effect of BiFeO_3_, as in other rare-earth doped bismuth ferrite systems. 

## Figures and Tables

**Table 1:** Amounts of precursors for Bi_1−x_Eu_x_FeO_3_ sol-gel synthesis.

	x = 0	x = 0.05	x = 0.10	x = 0.15	x = 0.20
Bi(NO_3_)_3_·5H_2_O	1.4554 g	1.3826 g	1.3098 g	1.2371 g	1.1643 g
Eu(NO_3_)_3_·5H_2_O	0 g	0.0643 g	0.1285 g	0.1928 g	0.2571 g
Fe(NO_3_)_3_·9H_2_O	1.2121 g	1.2121 g	1.2121 g	1.2121 g	1.2121 g
2-methoxyetanol	125 mL	125 mL	125 mL	125 mL	125 mL
Acetic acid	125 mL	125 mL	125 mL	125 mL	125 mL

**Table 2:** TG-DSC effects corresponding to Bi_1−x_Eu_x_FeO_3_ xerogels.

x = 0		x = 0.05		x = 0.10		x = 0.15		x = 0.20	
T (°C)	Mass Loss (%)	T (°C)	Mass Loss (%)	T (°C)	Mass Loss (%)	T (°C)	Mass Loss (%)	T (°C)	Mass Loss (%)
103.3	−4	103	−4.4	105.1	−3.7	108.3	−4.7	104.5	−4.7
214.1	−18.2	218.4	−26.5	221.6	−24.6	206.7	−12.4	208.7	−13.4
275.9	−14	278.0	−5.5	282.1	−8.7	239.2	−11.4	240.3	−11.1
413.5	−2.4	419.9	−2	422.2	−2.1	269.9	−10.8	277.9	−10.8
						427.8	−2.2	391.9	−2.7

**Table 3:** Agreement indices from Rietveld refinement for Bi_1−x_Eu_x_FeO_3_ calcined powders.

Agreement Indices	x = 0	x = 0.05	x = 0.10	x = 0.15	x = 0.20
R_exp_	6.1381	6.4479	6.4451	6.3596	6.4065
R_p_	5.5304	5.0238	6.7391	8.6136	8.7802
R_wp_	7.1636	6.4951	8.9802	11.6540	11.6193
χ^2^	1.3621	1.0147	1.9414	3.3581	3.2894

**Table 4:** Mössbauer parameters for x = 0 and x = 0.20 samples.

	IS (mm/s)	ΔEq (mm/s)	H_hf_ (T)
x = 0	0.335	0.179	48.90
x = 0.20	0.335	−0.054	48.89
SE	±0.001	±0.002	±0.02

**Table 5:** M_s_, M_r_ and H_c_ for Bi_1−x_Eu_x_FeO_3_ powders.

	x = 0	x = 0.05	x = 0.10	x = 0.15	x = 0.20
M_s_ (emu/g)	0.3529	1.6570	1.1089	0.7113	0.2968
M_r_ (emu/g)	0.0128	0.2287	0.0734	0.0551	0.0457
H_c_ (Oe)	51.290	101.160	69.883	91.156	36.815
